# Media Contribution to Dementia Awareness in Recruitment and Retention of Alzheimer’s Disease Diversity Genetic Cohorts‐ Alzheimer’s disease Sequencing Project in Africa

**DOI:** 10.1002/alz.091991

**Published:** 2025-01-09

**Authors:** Temitope Hannah Farombi, Oyedunni Arulogun, Michelle Nichols, Olorunyomi F Olorunsogbon, Timilehin Kumolu, Mayowa Ogunronbi, Taofeek Adedayo Sanni, Victoria Mutiso, Muhammed Uthman, Abdullateef Sule, Emmanuel Assey, Innocent Mwombeki, Joy Onoria, Godspower Chibuke Onunka, Benedict Tagoe, Selem K Melkamu, Ifeoma Adigwe, Adedoyin O Ogunyemi, Solomon Gyabaah, Goldie S. Byrd, Adesola Ogunniyi, Margaret A Pericak‐Vance, Rufus O. Akinyemi, Africa Dementia Consortium

**Affiliations:** ^1^ University College Hospital, Ibadan Nigeria; ^2^ University of Ibadan, Ibadan, Oyo Nigeria; ^3^ University of South Carolina, South Caroline, SC USA; ^4^ Institute for Advanced Medical Research and Training, College of Medicine, University of Ibadan, Ibadan, Oyo State Nigeria; ^5^ Afe Babalola University, Ado Ekiti, Ekiti Nigeria; ^6^ Africa Mental Health Research and Training Foundation, Nairobi Kenya; ^7^ University of Ilorin, Ilorin, Kwara Nigeria; ^8^ Ahmadu Bello University, Zaria, Kaduna Nigeria; ^9^ Kilimanjaro Christian Tanzania medical Center, Moshi, Moshi Tanzania, United Republic of; ^10^ Kilimanjaro Christian Tanzania Medical center, Moshi, Moshi Tanzania, United Republic of; ^11^ Louise Makerere University, Uganda, Uganda Uganda; ^12^ 7072 mulago hill, Kampala Uganda; ^13^ University of Ghana, Accra, Accra Ghana; ^14^ Addis Ababa University College of Health Sciences, Addis Ababa, Addis Ababa Ethiopia; ^15^ Nnamdi Azikwe University, Nsuka, Enugu Nigeria; ^16^ University of Lagos, Lagos Nigeria; ^17^ Komfo Anokye Teaching Hospital, Kumasi, Kumasi Ghana; ^18^ Maya Angelou Center for Health Equity, Wake Forest University School of Medicine, Winston‐Salem, NC USA; ^19^ College of Medicine, University of Ibadan, Ibadan, Oyo Nigeria; ^20^ 1501 NW 10th Avenue, Miami, FL USA; ^21^ Neuroscience and Aging Research Unit, Institute of Advanced Medical Research and Training, College of Medicine, University of Ibadan, Ibadan Nigeria

## Abstract

**Background:**

The escalating prevalence of dementia in Africa, propelled by rapidly ageing population, necessitates innovative approaches to raise awareness and address associated challenges. The prevalent misconception of dementia as a result of witchcraft or wizardry is a challenge, and the media acts as a key agent in dispelling such myths. By reaching divers audiences, the media reinforces the notion that dementia is not confined to Africa alone but it is a global concern. It also aids in overcoming the shame and stigma associated with dementia, encouraging individuals to seek consultation.

**Method:**

A comprehensive analysis of media initiatives used by the African Dementia Consortium (AfDC) during the World Alzheimer’s Month and subsequently was conducted. Digital Platforms (Instagram, LinkedIn, Facebook), print and traditional media (publications, journals, newspaper, radio and television stations) were examined for their effectiveness in achieving recruitment objectives, destigmatizing and promoting early diagnosis. Quality tools, such as cameras and recorders, were utilized to capture relevant information, while the outcome was evaluated based on their impact on target audience assessed by greater participation.

**Result:**

Media Platforms have proven instrumental in recruitment efforts by disseminating information that reaches diverse demographic groups in Africa. Through compelling visual and textual content, the media has played a key role in destigmatizing dementia, challenging prevalent misconceptions, and fostering a more inclusive understanding of the condition. In addition, media initiatives have contributed significantly to the promotion of early diagnosis by disseminating educational content that empowers individuals to recognize early signs and seek timely medical intervention.

**Conclusion:**

Both the digital and traditional media play vital roles in visually and textually representing dementia, correcting misconceptions, and fostering understanding. Allocating increased resources to media initiatives hold immense benefit for Africa, contributing to a more positive perspective on dementia and dispelling negative attitudes.

